# Aortic stiffness—Is kynurenic acid a novel marker? Cross-sectional study in patients with persistent atrial fibrillation

**DOI:** 10.1371/journal.pone.0236413

**Published:** 2020-07-31

**Authors:** Tomasz Zapolski, Anna Kamińska, Tomasz Kocki, Andrzej Wysokiński, Ewa M. Urbanska

**Affiliations:** 1 Chair and Department of Cardiology, Medical University of Lublin, Lublin, Poland; 2 Cardiological Health Resort Hospital, Nałęczów, Poland; 3 Chair and Department of Experimental and Clinical Pharmacology, Medical University of Lublin, Lublin, Poland; 4 Chair and Department of Experimental and Clinical Pharmacology, Laboratory of Cellular and Molecular Pharmacology, Medical University of Lublin, Lublin, Poland; University of Dundee, UNITED KINGDOM

## Abstract

**Objective:**

Although a number of modifiable and non-modifiable causes were implicated in arterial stiffness, its pathogenesis remains elusive, and very little is known about aortic elasticity in supraventricular arrhythmias. The potential role of disturbed kynurenine metabolism in the pathogenesis of cardiovascular disease has been recently suggested. Thus, we studied the correlations of aortic stiffness and echocardiographic parameters with biochemical markers and serum level of kynurenic acid (KYNA), an endothelial derivative of tryptophan, formed along the kynurenine pathway, among patients with atrial fibrillation (AF).

**Methods:**

Study cohort comprised 100 patients with persistent AF (43 females/57 males). Arterial stiffness index (ASI), structural and functional indices of left atrium (LA) and left ventricle (LV) were evaluated electrocardiographically. Biochemical analyses included the measurements of serum KYNA (HPLC) and of the selected markers of lipids and glucose metabolism, thyroid status, kidney function, inflammation and coagulation.

**Results:**

KYNA (β = 0.389, *P* = 0.029), homocysteine (β = 0.256, *P* = 0.40), total cholesterol (β = 0.814; *P* = 0.044), LDL (β = 0.663; *P* = 0.44), TSH (β = 0.262, *P* = 0.02), fT_3_ (β = -0.333, *P* = 0.009), fT_4_ (β = -0.275, *P* = 0.043) and creatinine (β = 0.374, *P* = 0.043) were independently correlated with ASI. ASI was also independently associated with LV end-systolic diameter (LVEDd; β = 1.751, *P* = 0.045), midwall fractional shortening (mFS; β = -1.266, *P* = 0.007), ratio mFS/end-systolic stress (mFS/ESS; β = -0.235, *P* = 0.026), LV shortening fraction (FS; β = -0.254, *P* = 0.017), and LA volume index (LAVI; β = 0.944, *P* = 0.022).

**Conclusions:**

In patients with AF, aortic stiffness correlated positively with KYNA, biochemical risk factors of atherosclerosis and with the indices of diastolic dysfunction of LV and LA. Revealed relationship between ASI and KYNA is an original observation, suggesting a potential role of disturbed kynurenine metabolism in the pathogenesis of arterial stiffening. KYNA, synthesis of which is influenced by homocysteine, emerges as a novel, non-classical factor associated with ASI in patients with AF.

## Introduction

The hemodynamic function of arteries, apart from the continuous transport of blood to the peripheral compartment, includes the transformation of pulsatile blood flow into a constant and stable stream. The compliance of vascular wall, defined as the relative change of vascular lumen area in proportion to the change of blood pressure, and distensibility, the proportion of compliance and initial pulse pressure, are important functional parameters of arteries [1, 2]. Arterial stiffness, the reciprocal of compliance and distensibility, reflects the deterioration of elasticity of large arteries [3, 4]. Importantly, aortic stiffness has emerged as a functional marker of atherosclerosis and a strong cardiovascular disease (CVD) risk factor. It is an independent predictor of cardiovascular morbidity and of all-cause mortality [5, 6]. Aortic stiffness is functionally quantified by various techniques, and aortic stiffness index (ASI) is an acknowledged method of its assessment [4]. Considering that arterial stiffness may be reversible under specific conditions, recognition of the underlying mechanisms and search for the novel therapeutic targets are of great interest.

So far, various modifiable and non-modifiable causes were implicated in the arterial stiffness. Genetic predisposition, inflammatory conditions, oxidative stress or hypertension-induced endothelial dysfunction were all implicated in the stiffening of arteries [7, 8]. The interplay between atherosclerosis and loss of arterial elasticity is supported by a number of experimental and clinical data. Arterial stiffness is linked with risk factors for atheroscelrosis, e.g. with dyslipidemia or metabolic syndrome [9, 10]. Furthermore, conditions such as hyperuricemia or hyperhomocysteinemia, possibly *via* generation of free radicals and increased inflammation, can promote the deterioration of structure and function of arterial wall [11–13].

Kynurenic acid (KYNA), a derivative of tryptophan formed along the kynurenine pathway, displays pleiotropic biological effects. KYNA is the only known endogenous antagonist of glutamatergic and α7 nicotinic receptors, as well as a ligand of G-protein coupled receptors 35 (GPR35), and of aryl hydrocarbon receptors (AHR) [14, 15]. As a result, KYNA exerts cytoprotective, neuromodulatory and anti-inflammatory effects [14, 15]. Kynurenine, the first stable product of tryptophan metabolism, can be transaminated either to KYNA, or, along another arm of the path, to the cytotoxic metabolites, such as 3-hydroxykynurenine, 3-hydroxyanthranilic acid or quinolinic acid [15]. The efficacy of kynurenine conversion depends on the expression and catalytic activity of the enzymes and on the various extra- and intracellular factors. The pro-inflammatory molecules, such as tumor necrosis factor-α (TNF-α), interleukins (ILs) or interferon-γ (IFN- γ), potently stimulate tryptophan breakdown and cause peripheral accumulation of the catabolites [15].

Altered homeostasis of KYNA was implicated in a number of pathological conditions, such as neurodegenerative, metabolic or cardiovascular disorders. Following the discovery of endothelial KYNA production [16, 17], the role of KYNA and other kynurenines in the pathogenesis of CVD has gained increasing attention. The endothelial compartment generates large quantities of KYNA, and this process is modified, among others, by the composition of ionic milieu, availability of oxygen and glucose, function of nitric oxide system or the extracellular concentration of homocysteine [16, 17]. In contrast, the toxic kynurenine metabolite, 3-hydroxykynurenine, was demonstrated to mediate the angiotensin II-induced apoptosis and to cause the dysfunction of endothelial cells [18]. Clinical studies revealed the correlation of intima media thickness with serum levels 3-hydroxykynurenine and quinolinate, in a cohort of patients with kidney insufficiency [19]. Furthermore, high serum levels of kynurenine and 3-hydroxykynurenine, but not of KYNA, were associated with an increased risk of acute coronary syndrome among aging subjects without prior history of CVD [20].

Atrial fibrillation (AF) remains one of the leading causes of morbidity and mortality among elderly patients. It is crucial to identify the factors promoting AF and to establish novel strategies aimed to evaluate the risk of AF occurrence. Approx. 50% of patients with AF suffer from the concomitant left ventricular diastolic dysfunction (LVDD). LVDD is the major factor contributing to the development of heart failure with preserved ejection fraction (HFpEF). Both conditions are closely related by the underlying pathology [21, 22]. In elderly AF patients with preserved systolic function, LV performance is directly associated with arterial stiffness [23]. Stiffening of arteries may increase the risk of AF through its impact on LV hypertrophy and resulting left atrium dysfunction [24]. This, in turn, may influence atrium remodeling and the incidence of arrhythmia. Furthermore, ASI genetic risk score is related with the incident AF [25]. The inflammatory component is one of the major factors connecting LVDD, left atrial remodeling and vascular changes such as fibrosis, endothelial dysfunction and arterial stiffening [26, 27].

Based on simultaneously performed hemodynamic-electrocardiographic analyses, we have previously shown the correlation between aortic stiffness and diastolic dysfunction of LV [28]. An increase of central aortic pressure, due to its reduced elasticity, was linked with higher end-diastolic pressure in LV. This, in the absence of mitral stenosis, was mirrored by the rise of LA volume, manifested by an increase of LA volume index (LAVI) and an increased pressure within LA [28]. Increase of LA volume is an important factor contributing to the induction of AF [25]. The mechanisms underlying the 'heart-vessel coupling disease', i.e. the interrelated stiffness of heart and arteries, are still poorly recognized [29]. Demonstrated associations between ASI, LAVI and parameters of systolic and diastolic function of LV, support the concept of ASI and LAVI being important markers of the heart-vessel coupling disease [28].

Considering that KYNA displays cytoprotective and anti-inflammatory properties, we have aimed to evaluate its potential relationship with arterial stiffness among patients with persistent AF. Echocardiographic studies combined with the biochemical analyses of serum KYNA, homocysteine, indicators of lipid metabolism, markers of myocardial, thyroid and kidney function and parameters of blood coagulation were performed.

## Materials and methods

### Study population

The studied cohort comprised 100 patients, age 69.1±7.8 years, including 43 women, age 70.1±8.34 years, and 57 men, age 68.3±7.5, admitted to the Chair and Department of Cardiology during the years 2017–2018 and diagnosed with clinically persistent AF. Patients were enrolled consecutively, unless exclusion criteria were applied. Exclusion criteria were: heart failure (NYHA class II or more), previous myocardial infarction or stroke, angiographically confirmed ischemic heart disease, hypertension stage 2 or 3, diabetes mellitus or renal failure. Patients were receiving the following drugs: ACE-inhibitors (65%), sartans (20%), calcium- channel blockers (35%), -blockers (75%), amiodarone (55%), propafenone (45%), statins (45%), warfarin (35%), dabigatran (25%) and rivaroxaban (40%). The study protocol conforms to the ethical guidelines of the 1975 Declaration of Helsinki. Written informed consent was obtained from all participants and the studies were approved by the Ethical Committee of Medical University of Lublin (KE-0254/27/2013).

### Echocardiography

The transthoracic echocardiography in motion mode (M-mode) was performed under the control of two-dimensional (2D) imaging in the parasternal longitudinal axis, using a 2.5–3.5 MHz probe working with Sonos 5500 and 7500 (Philips, Andover, MA, USA) echocardiographic units. An experienced cardiologist, not familiar with the clinical status of the patient, was carrying out the examinations. All of the measurements were performed according to the guidelines of American and European Echocardiographic Societies [30, 31], as described previously [11].

To assess the parameters of the ascending aorta, the ultrasound scan was performed at 3 cm above the aortic valve leaflet coaptation. The following aortic parameters were recorded: *aortic maximal diameter* (Ao_max_ [mm]), corresponding to the aortic systolic dimension, measured at the moment of full opening of the aortic valve and *aortic minimal diameter* (Ao_min_ [mm]), corresponding to the aortic diastolic dimension, measured at the peak of the QRS complex, established by simultaneously recorded electrocardiogram.

Left atrium (LA) measurements were performed when ultrasound beam was passing through coaptation of aortic valve leaflets; measurement was registered in M-mode, during end-systolic phase, right before opening of mitral valve: *LA maximal diameter*–LA_max_ [mm].

Left ventricle (LV) measurements registered in M-mode were performed when ultrasound beam was passing just under leaflets of mitral valve, and included: *interventricular septum systolic diameter*–IVSSd, [mm], *interventricular septum diastolic diameter*–IVSDd [mm], *posterior wall systolic diameter*–PWSd [mm], *posterior wall diastolic diameter*–PWDd mm, *LV end-systolic diameter*–LVESd [mm], and *LV end-diastolic diameter*–LVEDd [mm].

Four chamber apical view (4-CH) was used to measure: *LA short {medial-lateral} maximal diameter–*LA_shortmax_ [mm] during end-systolic phase, just before opening of mitral valve and *LA longitudinal maximal diameter {long axis}*–LA_longmax_, [mm] during end-systolic phase, just before opening of mitral valve.

The above planar parameters served for calculations of volumetric and functional parameters of aorta, LA and LV. The following aortic parameters were calculated:

- *aortic strain*–AS, [%]); AS = (Ao_max_−Ao_min_)/Ao_min_,
- *aortic distensibility*–AD, [cm^2^/dyn^-1^10^−6^]); AD = [2 x (Ao_max_−Ao_min_)]/[(Ao_min_ x PP)]; PP was calculated form systolic blood pressure (SBP) and diastolic blood pressure (DBP) measured simultaneously on right brachial artery while evaluating aortic parameters,
- *aortic stiffness index–*ASI, [n]); ASI = log {[SBP/DBP/(Ao_max_−Ao_min_)]/Ao_min_}; SBP and DBP were measured simultaneously on right brachial artery while evaluating aortic parameters.

The following volumetric parameters of LA were calculated:

- *LA maximal volume*–LAV_max_, [mL]); LAV_max_ = π/6 x (LA_max_ x LA_shortmax_ x LA_longmax_).
- *LA volume index*–LAVI, mL/m^2^); LAVI = LAVmax/m^2^; LAV adjustment based on the body surface:

The following indices of LV structure and function were calculated:

- *LV end-systolic volume*–LVESV, [mL]); according to Teichholz formula: LVESV = [7/(2,4 + LVESd)] x [LVESd]^3^,
- *LV end-diastolic volume—*LVEDV, [mL]); according to Teichholz formula: LVEDV = [7/(2,4 + LVEDd)] x [LVEDd]^3^,
- *LV stroke volume*–SV, [mL]); SV = LVEDV–LVESV,
- *stroke index*–SI, [mL/m^2^]); SI = SV/BSA,
- *cardiac output*–CO, [L/min]); CO = SV x HR,
- *cardiac index*–CI, [L/min/m^2^]; CI = CO/BSA,
- *LV ejection fraction*–EF, [%]); EF = [(LVEDV–LVESV)/LVEDV] x 100,
- *LV shortening fraction*–FS, [%]); FS = [(LVEDd–LVESd)/LVEDd] x 100,
- *end-systolic stress*–ESS, 10^3^dyn/cm^2^); according to the formula: ESS = 0,334 x SBP x LVESd/PWSd x (1 + PWSd/LVESd); SBP measured simultaneously on right brachial artery while evaluating aortic parameters
- *midwall fractional shortening*–mFS, %); according to the formula: mFS = (LVEDd + PWSd/2 + IVSSd/2)–(LVESd + Hs/2)/(LVEDd + PWSd/2 + IVSSd/2) x 100; where Hs = IVSSd + PWSd,
- *ratio* mFS/ESS, n,
- *LV mass*–LVM, g; according to the formula: LVM = 1,04 x (IVSDd + PWDd + LVEDd)^3^–13,6 g
- *LV mass index*–LVMI, g/m^2^; according to the formula: LVMI = LVM/BSA.

*Left ventricle hypertrophy*–LVH was diagnosed when: LVMI > 131 g/m^2^ (males), LVMI > 100 g/m^2^ (females).

Doppler analysis served to measure the flow through mitral valve. The following parameters were measured: *maximal velocity of early diastolic transmitral flow*–E [cm/s], *integral of early diastolic transmitral flow*–E_INTG_ [cm], and *deceleration time of early diastolic filling of left ventricle*–E_dcct_, [msec].

### Biochemical analyses

Blood samples were collected from a peripheral vein, between 8.00 and 10.00 a.m., and sera were stored at -72°C for further analyses. Homocysteine, total cholesterol (CHOL), low density lipoprotein cholesterol (LDL), high density lipoprotein cholesterol (HDL), triglycerides (TGC), fasting glucose, glycated haemoglobin 1C (HbA_1C_), thyroid-stimulating hormone (TSH), free triiodothyronine (fT_3_)_,_ free thyroxine (fT_4_), haemoglobin (Hb), red blood cells (RBC), white blood cells (WBC), platelets (PLT), haematocrit (HCT), aspartate transaminase (ASPAT), alanine transaminase (ALAT), high sensitive C-reactive protein (hs-CRP), fibrinogen, uric acid, creatinine, glomerular filtration rate (GFR), urea, myoglobin, creatine kinase (CK), CK-myocardial band (CK-MB) and brain natriuretic peptide (BNP) were analysed using ADVIA 1650, ADVIA 2120 and ADVIA Centaur analysers (Bayer–Bayer Health-Care Diagnostics, Tarrytown, New York, USA). Troponin T (TnT) was measured in full blood, using electrochemiluminescence method (Elecsys 2010; Roche Diagnostics GmbH, sensitivity 0.01 ng/mL).

### Quantification of KYNA

Serum KYNA levels were determined using high performance liquid chromatography (HPLC) method (Varian Pro Star 210 chromatograph with fluorescence detector; California, USA), as described previously [16]. On the day of analysis, samples of sera were defrosted and 0.5 ml of each sample was acidified with 100 μl of 50% trichloroacetic acid. Precipitated proteins were removed by centrifugation (12 000 rpm; 10 min). The supernatants were applied to the columns containing cation-exchange resin (Dowex 50 W^+^; 200–400 mesh; Sigma-Aldrich) prewashed with 1 ml of 0.1 N HCl. Columns were subsequently washed with 1 ml of 0.1 N HCl and 1 ml of ultra-pure water (Baker). KYNA was eluted with 2.5 ml of ultra-pure water (Baker). Eluted KYNA was subjected to HPLC (Varian Prostar HPLC isocratic system: ESA catecholamine HR-80, 3 μm particle size, C18 reverse-phase column) and quantified fluorimetrically (Varian ProStar fluorescence detector: excitation 344 nm, emission 398 nm). The mobile phase (50 mM sodium acetate, 250 mM zinc acetate and 4% acetonitrile, pH adjusted to 6.2) was pumped at a flow rate of 1.0 mL/min. The sensitivity was 150 fmol/injection (signal:noise ratio = 5). Standard calibration curve was constructed based on the signals obtained from external standards containing 0.2, 0.4, 0.6, 0.8 and 1 pmol of KYNA, and showed linearity of r^2^>0.999. KYNA concentration in the samples was expressed as nmol/L. HPLC reagents were purchased from Baker. The normal serum KYNA values in the population are in the range of 5 to 40.0 nmol/L, as determined in previous studies [32, 33].

### Statistical analyses

Statistical analyses were performed using Statistica software (version 6.0; StatSoft Inc.). Data are presented as mean values ± SD. Qualitative data are presented as values and percentage. Categorical variables were compared by chi-square test. Normal distribution of data was tested with the Shapiro-Wilk test, while homogeneity of variance was estimated with the Levene's test. The correlation between the parameters was described with Spearman's rank correlation coefficient. Significantly correlating parameters have been included in the creation of multiple stepwise regression analysis models. Multiple stepwise regression analysis was performed to estimate the potential influence of various factors on the ASI, AD and AS. The inflation variance coefficient was assessed for all parameters included in the multiple stepwise regression analysis. Thus, the value of this coefficient allowed to determine whether a given predictor was correlated with other predictors in the model. The lack of collinearity of predictors in the model was assumed in cases when the coefficient reached the value of ≤5, and the parameter was included in the analysis. However, when the value of the coefficient was >5, it indicated the possibility of collinearity between the predictors, consequently the parameter was excluded from the analysis. All models were adjusted for demographic and clinical confounders: age, sex, medications and hypertension. *R*^2^ describes the proportion or percentage of variance in the dependent variable explained by the variance in the independent variables together. An *R*^2^ of 1.00 indicates that 100% of the variation in the dependent variable is explained by the independent variables. Conversely, an *R*^2^ of 0.0 indicates the absence of variation in the dependent variable due to the independent variables. In this work, as the variables are not in the same unit of measures, a standardized regression coefficient *β*, was used. The *β* coefficients values show the direction, either positive or negative, and the contribution of the independent variable, relative to other independent variables, in explaining the variation of the dependent variable. Linear regression analyses for measurements from interval scale were performed with the use of Pearson correlation. *P*-value <0.05 was considered statistically significant.

## Results

Biochemical and echocardiographic characteristics of studied population are shown in Tables 1 and 2, respectively.

**Table 1:** The baseline biochemical characteristics of studied population.

Variable	Mean ± SD
KYNA [nmol/L]	23.7 (±12.8)
homocysteine [μmol/L]	19.99 (±6.18)
CHOL [mg/dL]	191.6 (±46.5)
LDL [mg/dL]	113.8 (±40.6)
HDL [mg/dL]	55.0 (±17.7)
TGC [mg/dL]	137.0 (±60.0)
glucose [mg/dL]	119.7 (±48.9)
HbA_1C_ [%]	6.53 (±1.28)
TSH [mU/L]	2.0 (±2.13)
fT_3_ [ng/L]	3.22 (±0,49)
fT_4_ [ng/L]	1.33 (±0.33)
Hb [g/dL]	14.12 (±1.40)
RBC [10^6^/μL]	4.85 (±0.44)
HCT [%]	42.69 (±4.07)
WBC [10^3^/μL]	7.0 (±1.8)
PLT [10^3^/μL]	250.2 (±73.2)
ASPAT [U/L]	32.8 (±26.5)
ALAT [U/L]	31.5 (±27.9)
hs-CRP [mg/L]	5.54 (±4.63)
fibrinogen [g/L]	3.58 (±0.92)
uric acid [mg/dL]	6.33 (±1.66)
urea [mg/dL]	41.9 (±13.5)
creatinine [mg/dL]	1.09 (±0.31)
GFR [ml/min/1,73m^2^]	67.34 (±18.45)
TnT [ng/mL]	0.019 (±0.020)
mioglobin [ng/mL]	62.71 (±28.17)
CK [U/L]	116.4 (±68.2)
CK-MB [U/L]	18.16 (±15.13)
BNP [pg/mL]	212.3 (±1204)

Data are presented as mean values ± SD.

**Table 2:** The baseline echocardiographic characteristics of studied population.

Variable	Mean ± SD
LVEDd [mm]	52.4 (±8.7)
LVESd [mm]	38.8 (±8.5)
IVSDd [mm]	12.2 (±2.2)
IVSSd [mm]	15.7 (±2.9)
PWDd [mm]	10.8 (±2.5)
PWST [mm]	15.9 (±2.1)
LVM [g]	291.2 (±113.5)
LVMI [g/m^2^]	146.2 (±50.5)
LVEDV [mL]	137.3 (±54.9)
LVESV [mL]	70.1 (±37.5)
LVSV [mL]	67.3 (±24.1)
CO [L/min]	5.1 (±1.1)
ESS [10^3^dyn/cm^2^]	161.1 (±33.9)
mFS [%]	20.3 (±4.6)
mFS/ESS [n]	0.137 (±0.055)
EF [%]	51.0 (±10.3)
FS [%]	26.6 (±6.2)
Ao_max_ [mm]	3.51 (±0.38)
Ao_min_ [mm]	3.28 (±0.41)
ASI [n]	3.65 (±1.52)
AD [cm^2^/dyn^-1^10^−6^]	5.96 (±3.73)
AS [%]	7.31 (±3.02)
E [cm/s]	90.7 (±24.7)
E_INTG_ [cm]	12.2 (±5.9)
E_dcct_ [msec]	126.4 (±29.0)
LA_max_ [mm]	46.21 (7.21)
LAVI [mL/m^2^]	40.82 (9.21)

Data are presented as mean values ± SD.

### Correlations between aortic echocardiographic parameters and biochemical markers

**Fig 1:**
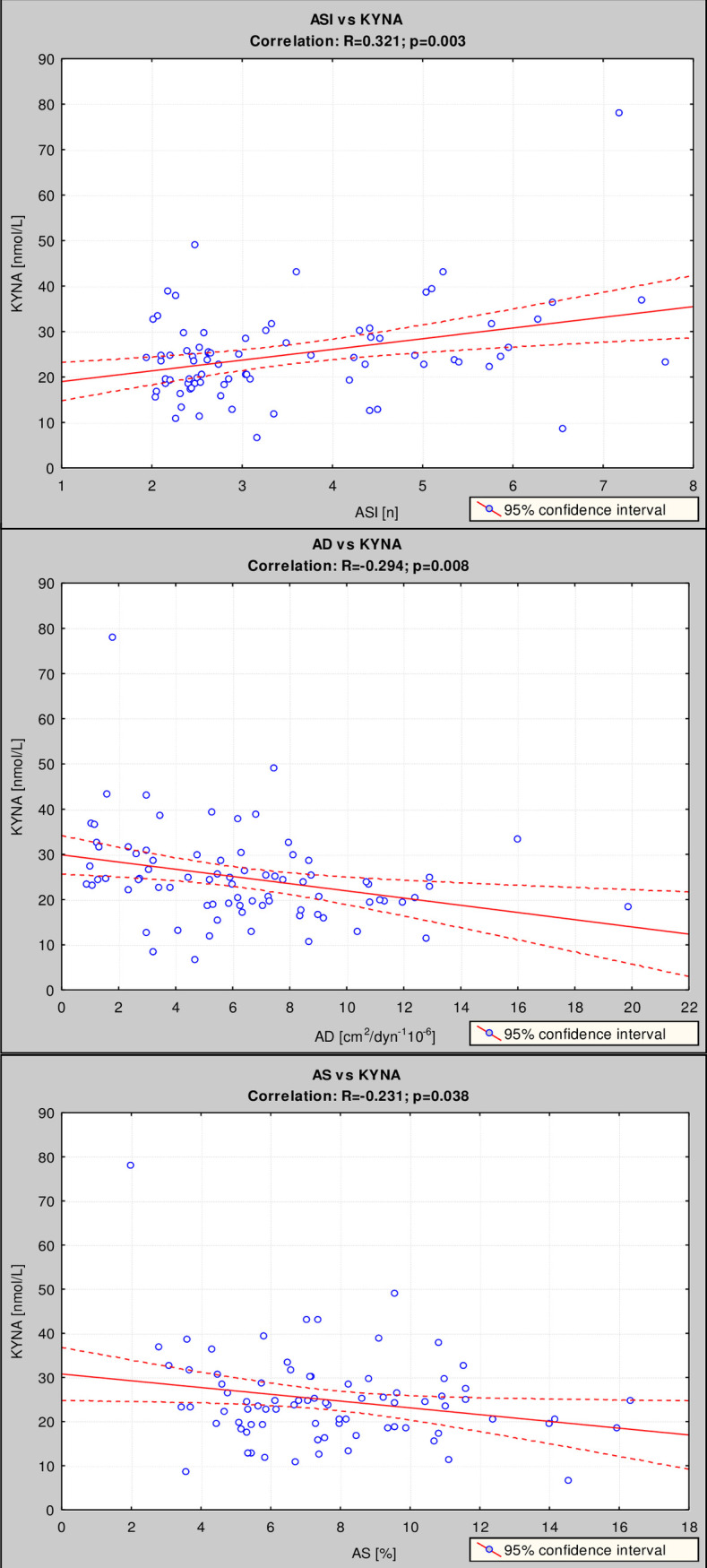
The correlation between ASI, AD and AS with serum KYNA level. Correlations between ASI (upper panel), AD (middle panel) and AS (lower panel) with serum KYNA level were estimated using Pearson’s method.

ASI correlated positively with serum KYNA (Fig 1), homocysteine, CHOL, LDL, TSH, hs-CRP, WBC, PLT, uric acid, creatinine, TnT and BNP (Table 3). ASI was linked negatively with serum fT3 and fT4 (Table 3). AD correlated negatively with serum KYNA (Fig 1), homocysteine, HCT, WBC, hs-CRP, fibrinogen, uric acid, urea and mioglobin (Table 3). AS correlated negatively with serum KYNA (Fig 1), homocysteine, WBC, uric acid and urea (Table 3).

**Table 3:** Correlations of ASI, AD and AS with KYNA and other biochemical variables in studied population.

Variable	Correlation with ASI		Correlation with AD		Correlation with AS	
	R	*P*-value	R	*P*-value	R	*P*-value
KYNA	**0.321**	**0.003**	**-0.294**	**0.008**	**-0.231**	**0.038**
homocysteine	**0.308**	**0.001**	**-0.319**	**0.003**	**-0.275**	**0.013**
CHOL	**0.235**	**0.018**	-0.15	0.181	0.095	0.398
LDL	**0.239**	**0.016**	-0.214	0.056	0.066	0.557
HDL	-0.073	0.467	0.156	0.165	0.026	0.812
TGC	0.152	0.13	-0.13	0.248	0.072	0.522
glucose	-0.021	0.832	-0.139	0.215	0.109	0.334
HbA_1C_	0.025	0.8	-0.181	0.106	0.1	0.377
TSH	**0.424**	**0.008**	0.008	0.938	0.15	0.181
fT_3_	**-0.246**	**0.011**	-0.153	0.174	-0.079	0.484
fT_4_	**-0.279**	**0.017**	-0.092	0.415	-0.178	0.113
Hb	0.051	0.608	-0.031	0.996	-0.033	0.765
RBC	-0.133	0.185	0.065	0.561	0.114	0.313
HCT	0.002	0.981	**-0.397**	**<0.001**	-0.062	0.584
WBC	**0.299**	**0.002**	**-0.245**	**0.028**	**-0.217**	**0.052**
PLT	**0.287**	**0.003**	-0.074	0.511	-0.083	0.459
ASPAT	0.039	0.694	-0.042	0.706	0.023	0.835
ALAT	0.045	0.649	-0.027	0.808	-0.015	0.892
hs-CRP	**0.265**	**0.007**	**-0.284**	**0.01**	0.124	0.271
fibrinogen	0.09	0.371	**-0.233**	**0.036**	-0.025	0.819
uric acid	**0.330**	**<0.001**	**-0.222**	**0.047**	**-0.228**	**0.041**
urea	0.067	0.507	**-0.267**	**0.016**	**-0.250**	**0.025**
creatinine	**0.309**	**0.001**	0.23	0.4	0.117	0.298
GFR	-0.186	0.063	**-0.231**	**0,038**	-0.08	0.48
TnT	**0.279**	**0.004**	-0.136	0.226	-0.091	0.417
mioglobin	0.171	0.088	**-0.226**	**0.043**	-0.189	0.093
CK	0.067	0.502	-0.038	0.732	-0.135	0.229
CK-MB	-0.118	0.238	0.16	0.155	0.177	0.116
BNP	**0.244**	**0.014**	-0.166	0.140	-0.189	0.091

R–correlation coefficient; Values in bold indicate statistical significance (*P*<0.05).

The multiple regression analysis showed that ASI was independently, positively correlated with serum KYNA, homocysteine, CHOL, LDL, TSH and creatinine, and negatively with fT3 and fT4 (Table 4). AD was independently, negatively correlated with serum KYNA (Table 5). AS was correlated negatively with serum homocysteine (Table 6).

**Table 4:** Independent biochemical parameters significantly related to ASI value.

Dependent variable	Independent variables		B	SE	*P-*value
ASI	KYNA	0.389	1.234	1.043	0.029
	homocysteine	0.256	0.063	0.033	0.040
	CHOL	0.814	0.026	0.013	0.044
	LDL	0.663	0.025	0.014	0.044
	TSH	0.262	0.188	0.079	0.020
	fT_3_	-0.333	-1.036	0.391	0.009
	fT_4_	-0.275	-1.273	0.618	0.043
	creatinine	0.374	1.837	1.082	0.043

Multiple regression analysis; model: R = 0.706, R^2^ = 0.499, *P*<0.05.—regression coefficient; B—non-standardized regression coefficient; SE—standard error; *P*—significance level.

**Table 5:** Independent biochemical parameters significantly related to AD value.

Dependent variable	Independent variables		B	SE	*P*-value
AD	KYNA	-0.367	-3.250	0.15	0.038
	fT3	-0.301	-2.280	1.37	0.031
	BNP	-0.325	-0.006	0.004	0.016

Multiple regression analysis; model: R = 0.666, R^2^ = 0.444, *P* = 0.246.—regression coefficient; B—non-standardized regression coefficient; SE—standard error; *P*—significance level.

**Table 6:** Independent biochemical parameters significantly related to AS value.

Dependent variable	Independent variables		B	SE	*P*-value
AS	homocysteine	-0.303	-0.135	0.092	0.048
	TSH	0.295	0.335	0.247	0.012

Multiple regression analysis; model: R = 0,650, R^2^ = 0.423, *P* = 0.34.—regression coefficient; B—non-standardized regression coefficient; SE—standard error; *P*—significance level.

### Correlations of ASI and echocardiographic parameters

ASI significantly correlated with LVEDd and LVESd, as well as with volumetric parameters of LV, i.e. with LVEDV, LVESV and LVSV (Table 7). Despite the fact that LV thickness did not correlate with ASI, calculated indices of LV hypertrophy, there was a significant correlation of ASI with LVM and LVMI (Table 7). ASI was associated negatively with the indicators of LV systolic function: mFS, mFS/ESS, SF and EF (Table 7). An indicator of systolic LV tension correlated positively with ASI (Table 7). No correlation between indicators of LV diastolic function calculated in Doppler analysis of flow has been observed.

**Table 7:** Correlations of ASI with echocardiographic parameters.

Parameter	ASI vs parameter	
	R	*P*-value
LVEDd	**0.404**	**0.00002**
LVESd	**0.400**	**0.00003**
IVSDd	0.086	0.393
IVSSd	0.095	0.343
PWDd	0.104	0.301
PWST	0.172	0.086
ESS	**0.475**	**0.000001**
mFS	**-0.235**	**0.018**
mFS/ESS	**-0.359**	**0.0002**
LVEDV	**0.404**	**0.00002**
LVESV	**0.400**	**0.00003**
LVSV	**0.291**	**0.003**
LVM	**0.346**	**0.0004**
LVMI	**0.292**	**0.003**
CO	0.111	0.268
EF	**-0.264**	**0.007**
FS	**-0.211**	**0.035**
Ao_max_	-0.046	0.642
Ao_min_	0.084	0.402
AD	**-0.685**	**<0.0001**
AS	**-0.658**	**<0.0001**
LA_max_	**0.574**	**<0.0001**
LA_shortmax_	**0.239**	**0.018**
LA_longmax_	**0.232**	**0.020**
LAVI	**0.469**	**<0.0001**
E	0.122	0.223
E_INTG_	0.050	0.620
E_time_	0.006	0.947
E_dcct_	0.031	0.756

R–correlation coefficient; Values in bold indicate statistical significance (*P*<0.05).

There was a significant correlation of ASI with an important marker of LA systolic dysfunction—LAVI (Table 7). Similarly, other parameters of LA function, such LA_max_, LA_shortmax_, LA_longmax_ correlated positively with ASI (Table 7). ASI correlated negatively with other parameters of aortic elasticity, i.e with AD and AS (Table 7).

The multiple regression analysis revealed independent, positive correlations of ASI with LVEDd and LAVI, and negative correlations of ASI with mFS, mFS/ESS, FS, AD and AS (Table 8).

**Table 8:** Echocardiographic parameters associated independently with ASI.

Dependent variable	Independent variables		B	SE	*P*-value
ASI	**LVEDd**	**1.751**	**0.306**	**0.207**	**0.045**
	**mFS**	**-1.266**	**-0.421**	**0.150**	**0.007**
	**mFS/ESS**	**-0.235**	**6.549**	**5.812**	**0.026**
	**FS**	**-0.254**	**-0.063**	**0.045**	**0.017**
	**LAVI**	**0.944**	**0.051**	**0.042**	**0.022**

Multiple regression model: R = 0.940, R^2^ = 0.884, *P*<0.001.—regression coefficient; B—non-standardized regression coefficient; SE—standard error; *P*—significance level. Values in bold indicate statistical significance (*P*<0.05).

## Discussion

Atherosclerosis is a process characterized by the plaque-forming degenerative changes within aorta and large arteries and by the considerably reduced vascular elasticity. The endothelial dysfunction, through its negative impact on the elastic properties of vasculature, plays a critical role in the progress of disease and the development of aortic stiffening [11, 34]. As a result of hemodynamic-volumetric feedback, the volume of LA increases and the probability of atrial arrhythmias is higher [11]. Endothelium produces large quantities of KYNA, in the process modulated *in vitro* by the serum level of homocysteine, a well-established marker of atherosclerosis [17]. Furthermore, clinical data demonstrate that KYNA level correlates positively with the level of homocysteine, at the early stage of stroke [32]. In here, we have evaluated the potential links of KYNA and other biochemical markers with the functional aspect of atherosclerosis, aortic stiffness, among patients with AF.

### Aortic stiffness and biochemical markers

Measurement of ASI is considered a valuable method allowing assessment of the cumulative influence of harmful factors on cardiovascular system [31]. In studied here cohort of patients, the functional marker of deteriorated arterial elasticity, ASI, correlated significantly with the serum levels of several compounds, including homocysteine, CHOL, LDL, markers of inflammation, such as WBC and hs-CRP, indicators of poor kidney function, such as creatinine and uric acid, and markers of compromised myocardial performance i.e. TnT and pro-BNP. The independent positive correlations with ASI were detected for KYNA, homocysteine, CHOL, LDL, creatinine and TSH. In contrast, the serum levels of thyroid hormones were associated negatively with ASI. Revealed relationship between ASI and KYNA is a novel observation, implying the role of disturbed kynurenine pathway in the pathogenesis of arterial stiffening.

Presented data are in agreement with previous findings underlying the contribution of ongoing inflammation, impaired renal filtration, and myocardial dysfunction to the development of aortic stiffness [1, 4, 5, 9]. Hypercholesterolemia and high LDL constitute important, modifiable risk factors of atherosclerosis, and their harmful influence on aortic elasticity is well substantiated [35, 36]. However, as suggested by a systematic review, the carotid-femoral pulse wave velocity, a measure of large arteries stiffness, is not independently associated with total cholesterol or LDL [37]. Considering that aortic stiffness remains an important marker of CVD risk [38], the measurement of ASI could be perceived as a better, cumulative predictor of cardiovascular events.

Although serum levels of thyroid hormones levels and TSH were within normal range, ASI was negatively associated with fT3 and fT4 levels, and positively with TSH. Similar correlation between arterial stiffness and high-normal TSH was shown in males with treatment-naïve hypertension [39], and in euthyroid healthy postmenopausal women [40]. The fT4 levels were inversely associated with arterial stiffness in euthyroid subjects [41]. Thus, our data support the concept on the important role of thyroid metabolism in preserving proper arterial elasticity, even in euthyroid subjects.

Reported link between ASI and homocysteine level corroborates previous studies. In studied here cohort of patients, the serum concentration of homocysteine (approx. 20 μmol/L) was elevated and in a range characteristic for the mild hyperhomocysteinemia (15–30 μmol/L). Others demonstrated that serum homocysteine is linked with aortic stiffness in hypertensive and normotensive subjects [42]. Molecular mechanisms underlying the homocysteine-induced deterioration of arterial elasticity are elusive, and seem to be a network of changes comprising disturbed wall structure and impaired endothelial function. Specifically, degradation of elastin, proliferation of vascular smooth muscles, increased collagen synthesis, activation of calcification and fibrosis, deficient nitric oxide formation, increased reactive species generation and endothelial damage, were associated with hyperhomocysteinemia [13, 43].

Positive correlation of ASI with uric acid, as observed in here, is consistent with the other reports. Higher serum homocysteine, uric acid and increased LA diameter were shown in persistent AF [44]. The association of hyperuricemia with arterial stiffness, impaired exercise capacity and high mortality was demonstrated in patients with HFpEF [45, 46]. Furthermore, serum uric acid may predict mortality risk in HFrEF, independently of chronic kidney disease [45, 46].

### Aortic stiffness and KYNA

Independent, positive correlation of serum KYNA with ASI is a novel observation. Up to our knowledge, the relationship between KYNA and the indices characterizing elastic properties of aorta, were not studied so far. KYNA is a biologically active metabolite of tryptophan, produced from its direct bioprecursor, kynurenine, along one arm of the kynurenine pathway. Second metabolic route of kynurenine generates mostly toxic compounds, such as 3-hydroxykynurenine or quinolinate [14, 15]. Noteworthy, KYNA, produced in large quantities by the endothelium [15], seems to be tightly linked with serum homocysteine levels. It was previously shown that homocysteine, possibly *via* its metabolite, S-adenosylhomocysteine, biphasically modulates the endothelial KYNA synthesis *ex vixo*, in rat aortic rings [16]. Low, micromolar concentrations of homocysteine, corresponding to values observed clinically in moderate hyperhomocysteinemia, increased the formation of KYNA. In contrast, high, milimolar levels potently inhibited KYNA synthesis [16]. In bovine endothelial cultures, KYNA diminished the homocysteine-evoked impairment of endothelial proliferation and reduced the migration and damage of endothelial cells [47]. In clinical scenario, serum KYNA levels positively correlate with homocysteine, as shown in the populations of healthy individuals, patients with stroke [32], or with uraemia [48].

Despite the increasing awareness about the role played by KYNA and other kynurenines in the pathogenesis of CVD and atherosclerosis, the potential link between disturbed KYNA levels and arterial stiffening was not evaluated so far. Considering that KYNA is able to counteract the homocysteine-induced injury of endothelial cells [47], it is tempting to view this kynurenine metabolite as a regulatory and protective compound. Indeed, KYNA was shown to increase the expression of metalloproteases and to suppress the synthesis of type-I collagen and fibronectin by fibroblasts [49], what further suggests beneficial role of KYNA in maintaining the arterial elasticity. Although in the studied here population, an average serum KYNA level reached ~24 nmol/L, and thus was within range found among control individuals (5–40 nmol/L) [32, 33], the local production of KYNA may easily achieve higher values. In fact, KYNA synthesis in human fibroblasts exposed to the pro-inflammatory cytokines increases by ten-fold [50]. Noteworthy, boosted KYNA synthesis may in turn reduce the inflammation, as demonstrated in various experimental models [51–53]. Furthermore, production of KYNA increases in the presence of nitric oxide donors, S-nitroso-N-acetylpenicillamine (SNAP), and 3-morpholinosydnonimine (SIN-1) [54]. It is probable, therefore, that the local interplay between homocysteine and KYNA is a regulatory endothelial mechanism aimed to limit the homocysteine-evoked toxicity. Indeed, KYNA, in contrast to the high levels of kynurenine and 3-hydroxykynurenine, was not associated with an increased risk of acute coronary syndrome among aging subjects [20]. Collectively, the accumulated data imply that KYNA plays the role of anti-inflammatory and cardioprotective compound.

Noteworthy, as recently reviewed, altered metabolism of kynurenine may contribute to the occurrence of vascular pathologies and stroke [55]. Following a stroke, the severity of infarct volume correlates with the levels of cytotoxic kynurenine derivatives [56]. Conversely, depletion of serum KYNA was described in the population of 81 patients suffering from the ischemic stroke [57]. Furthermore, experimental data suggest that therapeutic interventions aimed to increase KYNA levels may reduce the size of infarct and improve the behavioral consequences of stroke [55]. It is tempting to hypothesize that the link between improved neurological outcome after stroke and higher KYNA involves modulation of the aortic stiffness. Indeed, aortic stiffness plays harmful role in the acute phase of ischemic stroke through the reduction of collateral circulation what may limit the benefits of therapy [58]. Furthermore, ASI was demonstrated as a novel, independent predictor of hemorrhagic transformation among patients with ischemic stroke treated with thrombolysis [59].

### Aortic stiffness and echocardiographic parameters

Multiple regression analysis revealed the negative associations of ASI with echocardiographic parameters related to LV systolic function, mFS, mFS/ESS, EF and FS. Furthermore, positive correlation of ASI with the parameters of LV diastolic dysfunction, such as LVEDd and LAVI, was found. Furthermore, he indicator of systolic LV tension, ESS, correlated positively with ASI.

Diastolic dysfunction of LV, reflected by LVEDd, is commonly linked with an increased afterload, paralleling the loss of aortic elasticity. Structural changes of myocardium, mainly LV hypertrophy, are accompanying ESS [60]. Vicious cycle includes higher ESS, ventricular hypertrophy, increase of systolic and diastolic myocardial rigidity and further rise of workload. Progressing stiffness leads to the gradual decline of diastolic and systolic function of LV, with an increase of the end-diastolic pressure. This, in turn, increases the volume of LA. Indeed, in studied here group of patients, ASI was independently associated with the indices of LA volume, such as LA_max_ or LAVI. A significant relationship between the diameter of LA and the aortic stiffness, estimated from pulse wave velocity and humeral pulse pressure, was shown previously in a hypertensive population [61]. Stiff heart-artery coupling disease is a concept suggesting a tight correlation of arterial rigidity with systolic and diastolic stiffness of LV [29]. Ventricular-vascular stiffening impairs the coronary flow, contributes to the blood pressure irregularities and changes the endothelial function [62]. Interestingly, the arterial stiffness was a strong predictor of future AF among hypertensive patients, independently of their age, 24-hour pulse pressure or LA diameter [63].

Overall, our data bring novel information about the correlations of aortic stiffness with certain biochemical markers and with LV/LA rigidity in AF.

### Limitations

The number of enrolled patients was moderately high. Furthermore, the influence of applied pharmacotherapy, which may potentially affect the elastic properties of aorta, was not taken into consideration. Nevertheless, statins, known to influence ASI, were not used in the examined population of patients with AF.

## Conclusions

In chronic AF, aortic stiffness correlates positively with homocysteine, CHOL, LDL, TSH, hs-CRP, WBC, PLT, uric acid, creatinine, TnT and BNP levels and with the indices of diastolic dysfunction of LV and LA. KYNA has emerged as a novel, non-classical factor associated with ASI. We propose that the local interplay between homocysteine and KYNA may result in higher endothelial synthesis of KYNA and constitute a mechanism counteracting the homocysteine-induced toxicity.

Complex, reciprocal interactions between the endothelial function, levels of circulating metabolites and the stiffening of aorta require further research. There is a need of prospective studies aimed to determine whether ASI measurement alone, or in combination with serum homocysteine and KYNA, can be a predictor of therapy outcome in patients with persistent AF.

## Supporting information

S1 Data (ZIP)

## References

[1] AlGhatrif M, Lakatta EG. The conundrum of arterial stiffness, elevated blood pressure, and aging. Curr Hypertens Rep. 2015;17(2):12 10.1007/s11906-014-0523-z 25687599 PMC4524667

[2] Knutsen KM, Stugaard M, Michelsen S, Otterstad JE. M-mode echocardiographic findings in apparently healthy, nonathletic Norwegians aged 20–70 years. Influence of age, sex and body surface area. J Intern Med. 1989;225(2):111–5. 10.1111/j.1365-2796.1989.tb00049.x 2921591

[3] Glasser ST, Arnett DK, McVeigh GE, Finkelstein SM, Bank AJ, Morgan DJ, et al Vascular compliance and cardiovascular disease. A risk factor or a marker? Am Heart J. 1997;10 (10 Pt 1):1175–98. 10.1016/s0895-7061(97)00311-7 9370391

[4] Cavalcante JL, Lima JAC, Redheuil A, Al-Mallah MH. Aortic stiffness. Current understanding and future directions. J Am Coll Cardiol. 2011;57(14):1511–22. 10.1016/j.jacc.2010.12.017 21453829

[5] Tomiyama H, Ishizu T, Kohro T, Matsumoto C, Higashi Y. Longitudinal association among endothelial function, arterial stiffness and subclinical organ damage in hypertension. Int J Cardiol. 2018;253:161–6. 10.1016/j.ijcard.2017.11.022 29174285

[6] Bonarjee VVS. Arterial Stiffness: A Prognostic Marker in Coronary Heart Disease. Available Methods and Clinical Application. Front Cardiovasc Med. 2018;5:64 10.3389/fcvm.2018.00064 29951487 PMC6008540

[7] Raggi P, Boulay A, Chasan-Taber S, Amin N, Dillon M, Burke SK, et al Cardiac calcification in adult haemodialysis patients. A link between end-stage renal disease and cardiovascular disease? J Am Coll Cardiol. 2002;39(4):695–701. 10.1016/s0735-1097(01)01781-8 11849871

[8] Mozos I, Malainer C, Horbańczuk J, Gug C, Stoian D, Luca CT, et al Inflammatory Markers for Arterial Stiffness in Cardiovascular Diseases. Front Immunol. 2017;8:1058 10.3389/fimmu.2017.01058 28912780 PMC5583158

[9] Upala S, Wirunsawanya K, Jaruvongvanich V, Sanguankeo A. Effects of statin therapy on arterial stiffness: A systematic review and meta-analysis of randomized controlled trial. Int J Cardiol. 2017;227:338–41. 10.1016/j.ijcard.2016.11.073 27839806

[10] Zhao X, Wang H, Bo L, Zhao H, Li L, Zhou Y. Serum lipid level and lifestyles are associated with carotid femoral pulse wave velocity among adults: 4.4-year prospectively longitudinal follow-up of a clinical trial. Clin Exp Hypertens. 2018;40(5):487–94. 10.1080/10641963.2017.1384486 29035100

[11] Zapolski T, Wysokiński A, Książek A, Jaroszyński A. Aortic stiffness and left atrial volume index in patients on continuous ambulatory peritoneal dialysis: Role of endothelial dysfunction. Int J Cardiol. 201;162(3):253–6. 10.1016/j.ijcard.2012.06.111 22790190

[12] Zapolski T, Furmaga J, Jaroszyński A, Wysocka A, Rudzki, Wysokiński AP. The reverse remodeling of the aorta in patients after renal transplantation—the value of aortic stiffness index: prospective echocardiographic study. BMC Nephrology. 2017;18(1):33 10.1186/s12882-017-0453-5 28114900 PMC5260005

[13] Zhao J, Liu N, Chen J, Gu Y, Chen J, Yang K. Role of hyperhomocysteinemia and hyperuricemia in pathogenesis of atherosclerosis. J Stroke Cerebrovasc Dis. 2017;26(12):2695–9. 10.1016/j.jstrokecerebrovasdis.2016.10.012 28986198

[14] Urbańska EM, Chmiel-Perzyńska I, Perzyński A, Derkacz M, Owe-Larsson B. Endogenous kynurenic acid and neurotoxicity. In: Kostrzewa RS, editor. Handbook of Neurotoxicity. New York, NY: Springer; 2014 Volume 1, p. 421–53.

[15] Song P, Ramprasath T, Wang H, Zou MH. Abnormal kynurenine pathway of tryptophan catabolism in cardiovascular diseases. Cell Mol Life Sci. 2017;74(16):2899–2916. 10.1007/s00018-017-2504-2 28314892 PMC5501999

[16] Stążka J, Luchowski P, Wielosz M, Kleinrok Z, Urbańska EM. Endothelium-dependent production and liberation of kynurenic acid by rat aortic rings exposed to L-kynurenine. Eur J Pharmacol. 2002;448(2–3):133–7. 10.1016/s0014-2999(02)01943-x 12144932

[17] Stążka J, Luchowski P, Urbańska EM. Homocysteine, a risk factor for atherosclerosis. biphasically changes the endothelial production of kynurenic acid. Eur J Pharmacol. 2005;517(3):217–23. 10.1016/j.ejphar.2005.04.048 15961072

[18] Wang Q, Zhang M, Ding Y, Wang Q, Zhang W, Song P, et al Activation of NAD(P)H Oxidase by tryptophan-derived 3-hydroxykynurenine accelerates endothelial apoptosis and dysfunction in vivo. Circ Res. 2014;114(3):480–92. 10.1161/CIRCRESAHA.114.302113 24281189 PMC4104160

[19] Pawlak K, Myśliwiec M, Pawlak D. Kynurenine pathway—a new link between endothelial dysfunction and carotid atherosclerosis in chronic kidney disease patients. Adv Med Sci. 2010;55(2):196–203. 10.2478/v10039-010-0015-6 20439183

[20] Eussen SJM, Ueland PM, Vollset ST, Nygård O, Midttun O, Sulo G, et al Kynurenines as a predictors of acute coronary events in the Hordaland Health Study. Int J Cardiol. 2015;189:18–24. 10.1016/j.ijcard.2015.03.413 25885868

[21] Pai RG, Varadarajan P. Prognostic significance of atrial fibrillation is a function of left ventricular ejection fraction. Clin Cardiol. 2007;30(7):349–54. 10.1002/clc.20107 17674374 PMC6653692

[22] Kotecha D, Lam CSP, Van Veldhuisen DJ, Van Gelder IC, Voors A, Rienstra M. Heart failure with preserved ejection fraction and atrial fibrillation. J Am Coll Cardiol. 2016;68(20):2217–28. 10.1016/j.jacc.2016.08.048 27855811

[23] Fumagalli S, Migliorini M, Pupo S, Marozzi I, Boni S, Scardia A, et al Arterial stiffness and left ventricular performance in elderly patients with persistent atrial fibrillation. Aging Clin Exp Res. 2018;30(11):1403–8. 10.1007/s40520-018-0935-8 29569118

[24] Shaikh AK, Wang N, Yin X, Larson MG, Vasan RS, Hamburg NM, et al Relations of arterial stiffness and brachial flow-mediated dilation with new-onset atrial fibrillation: the Framingham heart study. Hypertension. 2016;68(3):590–6. 10.1161/HYPERTENSIONAHA.116.07650 27456517

[25] Zekavat SM, Roselli C, Hindy G, Lubitz SA, Ellinor PT, Zhao H, et al Genetic Link Between Arterial Stiffness and Atrial Fibrillation. Circ Genom Precis Med. 2019;12(6):e002453 10.1161/CIRCGEN.118.002453 31211625 PMC6582989

[26] Gumprecht J, Domek M, Lip GYH, Shantsila A. Invited review: hypertension and atrial fibrillation: epidemiology, pathophysiology, and implications for management. J Hum Hypertens. 2019;33(12):824–36. 10.1038/s41371-019-0279-7 31690818

[27] Boos CJ. Infection and atrial fibrillation: inflammation begets AF. Eur Heart J. 2020;41(10):1120–2. 10.1093/eurheartj/ehz953 31971996

[28] Zapolski T, Wysokiński A. Left atrium volume index is influenced by aortic stiffness and central pulse pressure in type 2 diabetes mellitus patients: A hemodynamic and echocardiographic study. Med Sci Monit. 2013;19(4):135–64. 10.12659/MSM.883818 PMC3628717 23458774

[29] Antonini-Canterin F, Carerj S, Di Bello V, Di Salvo G, La Carrubba S, Vriz O, et al On behalf of the Research Group of the Italian Society of Cardiovascular Echocardiography (SIEC): Arterial stiffness and ventricular stiffness: a couple of disease or a coupling disease? A review from the cardiologist’s point of view. Eur J Echocardiogr. 2009;10(1):36–43. 10.1093/ejechocard/jen236 18799479

[30] Patel M, Spertus J, Brindis R, Hendel R, Douglas P, Peterson E, et al ACCF proposed method for evaluating the appropriateness of cardiovascular imaging. J Am Coll Cardiol. 2005;46(8):1606–13. 10.1016/j.jacc.2005.08.030 16226195

[31] Laurent S, Cockroft J, Van Bortel L, Boutouyrie P, Giannattasio C, Hayoz D, et al On behalf of the European Network for Non-invasive Investigation of Large Arteries: Expert consensus document on arterial stiffness: methodological issues and clinical applications. Eur Heart J. 2006;27(21):2588–605. 10.1093/eurheartj/ehl254 17000623

[32] Urbańska EM, Luchowski P, Luchowska E, Pniewski J, Woźniak R, Chodakowska-Zebrowska M, et al Serum kynurenic acid positively correlates with cardiovascular disease risk factor, homocysteine: a study in stroke patients. Pharmacol Rep. 2006;58(4):507–11. 16963796

[33] Szymona K, Zdzisińska B, Karakuła-Juchnowicz H, Kocki, Kandefer-Szerszeń M, Flis M, et al Correlations of Kynurenic Acid, 3-Hydroxykynurenine, sIL-2R, IFN-α, and IL-4 with Clinical Symptoms During Acute Relapse of Schizophrenia. Neurotox Res. 2017;32(1):17–26. 10.1007/s12640-017-9714-0 28275903

[34] Zapolski T, Wysokiński A, Książek A, Jaroszyński A. Left atrial volume index and aortic stiffness index in adult hemodialysed patients—link between compliance and pressure mediated by endothelium dysfunction, a cross-sectional study BMC Cardiovasc Disord. 2012;12:100 10.1186/1471-2261-12-100 23122326 PMC3519803

[35] Pitsavos C, Toutouzas K, Dernellis J, Skoumas J, Skoumbourdis E, Stefanadis C, et al Aortic stiffness in young patients with heterozygous familial hypercholesterolemia. Am Heart J. 1998;135(4):604–8. 10.1016/s0002-8703(98)70274-1 9539474

[36] Kontopoulos AG, Athyros VG, Pehlivanidis AN, Demitriadis DS, Papageorgiu AA, Boudoulas H. Long–term treatment effect of atrovastatin on aortic stiffness in hypercholesterolaemic patients. Curr Med Res Opin. 2003;19(1):22–7. 10.1185/030079902125001290 12661776

[37] Cecelja M, Chowienczyk P. Dissociation of aortic pulse wave velocity with risk factors for cardiovascular disease other than hypertension. Hypertension 2009;54(6):1328–36. 10.1161/HYPERTENSIONAHA.109.137653 19884567

[38] Laurent S, Boutouyrie P, Asmar R, Gautier I, Laloux B, Guize L, et al Aortic stiffness is an independent predictor of all cause cardiovascular mortality in hypertensive patients. Hypertension. 2001;37(5):1236–1241. 10.1161/01.hyp.37.5.1236 11358934

[39] Kwon B, Roh JW, Lee SH, Lim SM, Park CS, Kim DB, et al A high normal thyroid-stimulating hormone is associated with arterial stiffness, central systolic blood pressure, and 24-hour systolic blood pressure in males with treatment-naïve hypertension and euthyroid. Int J Cardiol. 2014;177(3):949–56. 10.1016/j.ijcard.2014.09.200 25449506

[40] Lambrinoudaki I, Armeni E, Rizos D, Georgiopoulos G, Kazani M, Alexandrou A, et al High normal thyroid-stimulating hormone is associated with arterial stiffness in healthy postmenopausal women. J Hypertens. 2012;30(3):592–9. 10.1097/HJH.0b013e32834f5076 22227818

[41] Wang J, Zheng X, Sun M, Wang Z, Fu Q, Shi Y, et al REACTION Study Group. Low serum free thyroxine concentrations associate with increased arterial stiffness in euthyroid subjects: a population-based cross-sectional study. Endocrine. 2015;50(2):465–73. 10.1007/s12020-015-0602-1 25987347

[42] Vyssoulis G, Karpanou E, Kyvelou S-M, Adamopoulos D, Gialernios T, Gymnopoulou E, et al Associations between plasma homocysteine levels, aortic stiffness and wave reflection in patients with arterial hypertension, isolated office hypertension and normotensive controls. J Hum Hypertens. 2010;24(3):183–9. 10.1038/jhh.2009.50 19516272 PMC2845510

[43] Esse R, Barroso M, Tavares de Almeida I, Castro R. The Contribution of Homocysteine Metabolism Disruption to Endothelial Dysfunction: State-of-the-Art. Int J Mol Sci. 2019;20(4):E867 10.3390/ijms20040867 30781581 PMC6412520

[44] Shi D, Meng Q, Zhou X, Li L, Liu K, He S, et al Factors influencing the relationship between atrial fibrillation and artery stiffness in elderly Chinese patients with hypertension. Aging Clin Exp Res. 2016;28(4):653–8. 10.1007/s40520-015-0455-8 26386864

[45] Shimizu T, Yoshihisa A, Kanno Y, Takiguchi M, Sato A, Miura S, et al Relationship of hyperuricemia with mortality in heart failure patients with preserved ejection fraction. Am J Physiol Heart Circ Physiol 2015;309(7):H1123–9. 10.1152/ajpheart.00533.2015 26297226

[46] Filippatos GS, Ahmed MI, Gladden JD, Mujib M, Aban IB, Love TE, et al Hyperuricaemia, chronic kidney disease, and outcomes in heart failure: potential mechanistic insights from epidemiological data. Eur Heart J. 2011;32(6):712–20. 10.1093/eurheartj/ehq473 21199831 PMC3056205

[47] Wejksza K, Rzeski W, Okuno E, Kandefer-Szerszeń M, Albrecht J, Turski W. Kynurenic acid protects against the homocysteine-induced impairment of endothelial cells. Pharmacol Rep. 2009;61(4):751–6. 10.1016/s1734-1140(09)70130-6 19815960

[48] Pawlak K, Myśliwiec M, Pawlak D. Hyperhomocysteinemia and the presence of cardiovascular disease are associated with kynurenic acid levels and carotid atherosclerosis in patients undergoing continuous ambulatory peritoneal dialysis. Thrombosis Res. 2012;129(6):704–9. 10.1016/j.thromres.2011.08.016 21906785

[49] Poormasjedi-Meibod MS, Hartwell R, Kilani RT, Ghahary A. Anti-scarring properties of different tryptophan derivatives. PLoS One 2014;9(3):e91955 10.1371/journal.pone.0091955 24637853 PMC3956813

[50] Asp L, Johansson AS, Mann A, Owe-Larsson B, Urbanska EM Kocki T, et al Effects of pro-inflammatory cytokines on expression of kynurenine pathway enzymes in human dermal fibroblasts. J Inflamm. 2011;8:25. 10.1186/1476-9255-8-25 PMC3204223 21982155

[51] Maes M, Mihaylova I, Ruyter MD, Kubera M, Bosmans E. The immune effects of TRYCATs (tryptophan catabolites along the IDO pathway): relevance for depression and other conditions characterized by tryptophan depletion induced by inflammation. Neuro Endocrinol Lett. 2007;28(6):826–31. 18063923

[52] Lugo-Huitrón R, Blanco-Ayala T, Ugalde-Muńiz P, Carrillo-Mora P, Pedraza-Chaverrí J, Silva-Adaya D, et al On the antioxidant properties of kynurenic acid: free radical scavenging activity and inhibition of oxidative stress. Neurotoxicol Teratol. 2011;33(5):538–47. 10.1016/j.ntt.2011.07.002 21763768

[53] Małaczewska J, Siwicki AK, Wójcik RM, Turski WA, Kaczorek E. The effect of kynurenic acid on the synthesis of selected cytokines by murine splenocytes—in vitro and ex vivo studies. Cent Eur J Immunol. 2016;41(1):39–46. 10.5114/ceji.2016.58815 27095921 PMC4829820

[54] Luchowski P, Urbanska EM. SNAP and SIN-1 increase brain production of kynurenic acid. Eur. J. Pharmacol. 2007;563(1–3):130–3. 10.1016/j.ejphar.2007.02.044 17391664

[55] Colpo GD, Venna VR, McCullough LD, Teixeira AL. Systematic Review on the Involvement of the Kynurenine Pathway in Stroke: Pre-clinical and Clinical Evidence. Front Neurol. 2019;10:778 10.3389/fneur.2019.00778 31379727 PMC6659442

[56] Darlington LG, Mackay GM, Forrest CM, Stoy N, George C, Stone TW. Altered kynurenine metabolism correlates with infarct volume in stroke. Eur J Neurosci. 2007;26(8):2211–21. 10.1111/j.1460-9568.2007.05838.x 17892481

[57] Mo X, Pi L, Yang J, Xiang Z, Tang A. Serum indoleamine 2,3-dioxygenase and kynurenine aminotransferase enzyme activity in patients with ischemic stroke. J Clin Neurosci. 2014;21(3):482–6. 10.1016/j.jocn.2013.08.020 24412293

[58] Acampa M, Romano DG, Lazzerini PE, Leonini S, Guideri F, Tassi R, et al Increased Arterial Stiffness is Associated with Poor Collaterals in Acute Ischemic Stroke from Large Vessel Occlusion. Curr Neurovasc Res. 2018;15(1):34–8. 10.2174/1567202615666180326100347 29577862

[59] Acampa M, Camarri S, Lazzerini PE, Guideri F, Tassi R, Valenti R, et al Increased arterial stiffness is an independent risk factor for hemorrhagic transformation in ischemic stroke undergoing thrombolysis. Int J Cardiol. 2017;243:466–70. 10.1016/j.ijcard.2017.03.129 28747037

[60] Paulus W J Tschope C, Sanderson JE, Rusconi C, Flachskampf FA, Rademakers FE, et al How to diagnose diastolic heart failure: a consensus statement on the diagnosis of heart failure with normal left ventricular ejection fraction by the Heart Failure and Echocardiography Associations of the European Society of Cardiology. Eur Heart J. 2007;28(20):2539–50. 10.1093/eurheartj/ehm037 17428822

[61] Lantelme P, Laurent S, Besnard C, Bricca G, Vincent M, Legedz L, et al Arterial stiffness is associated with left atrial size in hypertensive patients. Arch Cardiovasc Dis. 2008;101(1):35–40. 10.1016/s1875-2136(08)70253-5 18391871

[62] Kass DA. Ventricular arterial stiffening. Integrating the pathophysiology. Hypertension. 2005;46(1):185–93. 10.1161/01.HYP.0000168053.34306.d4 15911741

[63] Cremer A, Lainé M, Papaioannou G, Yeim S, Gosse P. Increased arterial stiffness is an independent predictor of atrial fibrillation in hypertensive patients J Hypertens. 2015;33(10):2150–5. 10.1097/HJH.0000000000000652 26431194

